# Functional Characterization and Transcriptional Regulation of Olive Tocopherol Methyltransferase Responsible for the Vitamin E Content of Virgin Olive Oil

**DOI:** 10.1021/acs.jafc.6c07768

**Published:** 2026-08-24

**Authors:** Cristina Muñoz-Ocaña, Isabel Narváez, M. Dolores Sicardo, M. Luisa Hernández, Wenceslao Moreda, José M. Martínez-Rivas

**Affiliations:** Department of Biochemistry and Molecular Biology of Plant Products, 54444 Instituto de la Grasa (IG), CSIC, Seville 41013, Spain

**Keywords:** Olea europaea, olive fruit, α-tocopherol, TMT, functional identification, gene expression, abiotic stresses

## Abstract

The development of novel olive cultivars with enhanced vitamin E levels in their virgin olive oil is a major goal in olive breeding programs. This study reports the cloning and characterization of an olive cDNA, which encodes tocopherol methyltransferase (TMT) that catalyzes the last reaction of tocopherol biosynthesis. Sequence analysis and bacterial functional expression confirmed that the gene encodes an active TMT enzyme. Expression profiling across different olive tissues and cultivars revealed a cultivar-dependent spatial and temporal regulation of TMT transcripts. When combined with tocopherol profiling, the data suggest that *OeTMT* is implicated in the biosynthesis of the α-tocopherol present in olive mesocarp and, consequently, in the olive oil. Additionally, the α-tocopherol biosynthesis in the olive mesocarp appears to be transcriptionally regulated in response to environmental factors, including water status, temperature, light, and wounding, indicating that *OeTMT* could play a role in the response to abiotic stress.

## Graphical abstract

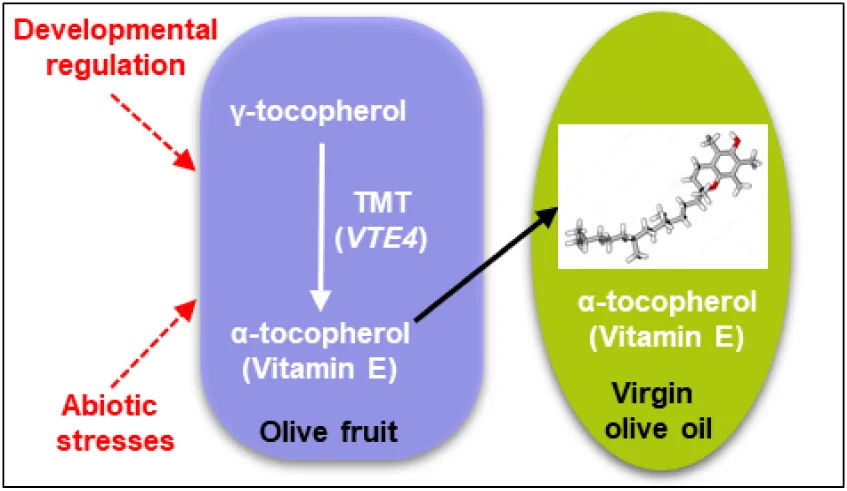

## Introduction

Virgin olive oil (VOO) is a juice derived solely through the mechanical extraction of olive fruit, without additives or preservatives. Its production and consumption have significantly increased due to its superior sensory qualities and remarkable nutritional benefits,^1^ largely determined by the compounds it contains. VOO is primarily composed of triacylglycerols as major compounds (≥98%) along with a varied array of chemical components named “minor compounds” (≤2%).^2^ Among these minor constituents are tocopherols, with four chemical forms identified in olives. Tocopherols have a simple structure consisting of a polar chromanol ring with a lipophilic saturated polyprenyl side chain.^3^ The four tocopherol variants are distinguished by the number of methyl groups on the chromanol ring: α- has three, β- and γ-have two, and δ-tocopherol has one.^4^

Vitamin E, discovered in 1922, is the collective name for a group of fat-soluble compounds with distinct antioxidant activities and has been extensively studied in mammals. Beyond its essential function in reproduction, vitamin E provides health benefits in humans,^5^ including protection against chronic and neurological diseases linked to oxidative stress, such as cancer and atherosclerosis.^6,7^ Among tocopherols, α-tocopherol possesses the maximum biological activity owing to its high affinity for the α-tocopherol transfer protein that transports it into the bloodstream.^8^ Humans must obtain vitamin E from dietary sources like nuts, seeds, grains, plant oils, fruits, and green vegetables.^9^ Specifically, sunflower and wheat germ oils are rich in α-tocopherol, whereas γ-tocopherol predominates in soybean, corn, and rapeseed oils.^10^ Consequently, enhancing vitamin E content, particularly α-tocopherol, in vegetable oils represents a key breeding target.^11^

In plants, tocopherol content and composition vary by tissue, with green tissues typically rich in α-tocopherol.^12^ Many seeds mainly accumulate γ-tocopherol, while α-tocopherol is abundant in the leaf,^13^ though exceptions exist—namely, sunflower seeds, which predominantly contain α-tocopherol.^14^ α-Tocopherol is mostly localized in the inner envelope and thylakoid membranes of chloroplasts, as well as occasionally in plastoglobuli.^13^

In photosynthetic organisms, tocopherols primarily serve as nonenzymatic antioxidants, protecting lipids from oxidation by quenching singlet oxygen and scavenging lipid peroxy radicals during stress.^15^ Recent studies also implicate tocopherols in plant development, stress responses, membrane stability, gene regulation, and intracellular signaling.^11,16,17^

Tocopherol biosynthesis occurs in plastids, beginning with the condensation of homogentisic acid (HGA) and phytyl diphosphate (PDP), which is catalyzed by homogentisate phytyltransferase (HPT), resulting in the formation of 2-methyl-6-phytyl-1,4-benzoquinol (MPBQ).^4,18^ MPBQ can be methylated by MPBQ methyltransferase (MPBQ MT) to form 2,3-dimethyl-6-phytyl-1,4-benzoquinol (DMPBQ). Both MPBQ and DMPBQ are cyclized by tocopherol cyclase (TC) into δ- and γ-tocopherols, respectively. Finally, tocopherol methyltransferase (TMT) methylates δ- and γ-tocopherols into β- and α-tocopherols, respectively.^11^ Thus, TMT catalyzes the last reaction of tocopherol biosynthesis, controlling the ratio of β- and α- to δ- and γ-tocopherols.

The *TMT* gene (*VTE4*) was first cloned from *Arabidopsis* and *Synechocystis*.^19^ Leaves of the *Arabidopsis* *vte4* mutant lack α- and β-tocopherols and accumulate γ-tocopherol and some δ-tocopherol.^20^ Conversely, *AtTMT* overexpression in *Arabidopsis* leaves increased α- and total tocopherol levels,^21^ highlighting TMT activity as a limiting factor for α-tocopherol biosynthesis in the leaf.^4^ Similarly, seed-specific *TMT* expression completely converts γ-tocopherol to α-tocopherol in *Arabidopsis* and soybean.^19,22^ Since most seeds mainly accumulate γ-tocopherol, TMT activity may represent a bottleneck for α-tocopherol biosynthesis in seeds.^11^

Vitamin E enrichment in staple crops, mostly oilseeds, represents a primary objective in plant breeding owing to its positive impacts on human health and oil quality.^23^ Specifically, key genes within the vitamin E biosynthetic pathway have been overexpressed, either singly or in combination, in plants such as soybean and rapeseed.^11^ Achieving a major increase in total vitamin E accumulation is still difficult, however, given the pathway’s complexity and regulatory mechanisms.

In VOO, the total tocopherol amount varies from 90 to 1400 mg kg^–1^, with α-tocopherol comprising over 96%, while γ- and β-tocopherols represent much lower percentages of around 2.5% and 1%, respectively.^24^ Nearly all VOO tocopherols originate from the olive fruit mesocarp, with minor seed contributions.^25^ α-Tocopherol in olive mesocarp ranges between 122 and 521 mg kg^–1^.^26^ Tocopherol content and composition of VOO are influenced by cultivar, fruit maturation stage, and agroclimatic factors.^26,27^ Unlike phenolic secoiridoids, tocopherols remain largely stable during oil extraction, with a direct transfer from the fruit to the VOO. Nevertheless, some loss occurs during the centrifugation step in the added water because of their amphipathic nature.^28^

Recent breeding efforts aim to develop new olive cultivars yielding oils with improved functional quality, including higher vitamin E content.^24^ Achieving this requires identifying molecular markers linked to high vitamin E content. Nevertheless, unlike seeds or leaves, molecular investigations on the tocopherol biosynthetic pathway in oil fruits are scarce. Among the four genes involved in tocopherol biosynthesis, only *HPT* and *MPBQ MT* have been cloned and characterized in olive recently by our group.^29^ The objectives of the present work focus on isolating and functionally characterizing the olive *TMT* gene, analyzing tocopherol content and gene expression throughout fruit development and maturation in different cultivars, and examining TMT’s role in α-tocopherol biosynthesis and abiotic stress responses in the olive mesocarp.

## Materials and Methods

### Plant Material and Stress Treatments

Olive (*Olea europaea* L.) trees of the primary oil-producing cultivars, Picual and Arbequina, were cultivated at the Instituto de la Grasa experimental orchard (Seville, Spain) using drip irrigation and fertirrigation from full bloom to fruit ripening. Additional cultivars with low (“Klon-14”, “Abou Kanani”) and high (“Dokkar”, “Piñonera”) tocopherol content were grown under standard conditions at the Worldwide Olive Germplasm Bank of Córdoba-ESP046, located at IFAPA “Alameda del Obispo” (Córdoba, Spain).

Olive tissues, including young leaves, young drupes, and developing seeds and mesocarp at distinct stages during the development and ripening of olive fruit, were sampled as previously described.^29^

Water deficit experiments were conducted in a super high-density Arbequina orchard close to Seville using two different regulated deficit irrigation (RDI) treatments (30RDI and 60RDI), with mesocarp samples collected at different developmental and maturation stages according to Hernández et al.^30^

Stress treatments were performed as described previously.^31^ Branches from olive trees of Picual and Arbequina cultivars, holding approximately 100 turning-stage (28 WAF) fruits were incubated in growth chambers under 12 h light/12 h dark cycles at 25 °C (standard conditions). For stress assays, conditions were modified accordingly, with zero time set 2 h after light onset to maintain the natural photoperiod.

In all cases, olive samples were immediately frozen in liquid nitrogen after harvest and stored at −80 °C.

### Isolation of Olive Tocopherol Methyltransferase Full-Length cDNA Clone and Sequence Analysis

A putative olive *TMT* sequence was found in the olive transcriptome^32^ and genome^33^ via tblastn and *Arabidopsis* TMT (At1g64970) as the query. Specific primers based on this sequence were used for PCR amplification with ACCUZYME DNA polymerase (Bioline, Spain), which has proofreading activity, together with an aliquot of a 13 WAF olive (cv. Picual) fruit cDNA library as the template. An amplified fragment was cloned in the pSpark I vector (Canvax, Spain) and sequenced bidirectionally (STAB vida, Portugal). DNA sequence analysis was carried out as previously reported.^34^ The cDNA sequence corresponding to olive *TMT* (*OepTMT*) has been deposited in GenBank with the accession number PX970942.

### Expression Analysis of Olive Tocopherol Methyltransferase Gene

Total RNA was extracted from frozen olive tissue (1.5 g) as previously described.^35^ RNA quality assessment, DNA removal, and cDNA synthesis were performed as described earlier.^36^

Gene expression was measured by quantitative real-time PCR (qRT-PCR) using a CFX Connect real-time PCR System and iTaq Universal SYBR Green Supermix (BioRad, California, USA), as previously reported.^31^ Specific primers for *OeTMT* amplification were designed with the Primer3 program (http://bioinfo.ut.ee/primer3/) and the Gene Runner program (Table S1), and the olive ubiquitin2 gene (*OeUBQ2*, AF429430) served as an endogenous control.^36^ Expression was calibrated relative to 12 WAF “Picual” mesocarp for developmental studies, 13 WAF “Arbequina” mesocarp FI treatment for water deficit experiments, and zero time for stress treatments. Relative expression was calculated using the 2^–ΔΔCt^ method.^37^ Data are means ± SD of three biological replicates, with two technical replicates each per 96-well plate.

### Functional Expression of Olive Tocopherol Methyltransferase Gene in *Escherichia coli*

The olive *TMT* coding sequence, excluding the N-terminal chloroplast transit peptide (81 amino acids), was PCR-amplified using ACCUZYME DNA polymerase and specific primers containing restriction sites for directional ligation (Table S2). The amplified product was double-digested and ligated into the pET45b(+) bacterial expression vector (Novagen, Germany) under the control of the IPTG-inducible T7*lac* promoter. Constructs were sequence-verified and transformed into *E. coli* strain BL21 (DE3) (Novagen, Germany) and selected on LB ampicillin plates. Six milliliter of LB medium containing ampicillin was inoculated with a single colony and incubated at 37 °C until A_600_ reached 0.3–0.5. Subsequently, 100 mL of LB medium with ampicillin was inoculated with all the preculture and incubated at 37 °C. When the A_600_ of the culture was 0.6, gene expression was induced with 10 mM IPTG, and the culture was further grown for 20 h at 22 °C. *E. coli* cells were centrifuged for harvesting at 2,500*g* for 10 min at 4 °C, washed with 50 mM phosphate buffer (pH 7.5), frozen in liquid nitrogen, and stored at −80 °C.

For crude extract preparation, the frozen cell pellet was defrosted and resuspended in 5 mL of extraction buffer [10 mM HEPES pH 7.8, 5 mM dithiothreitol (DTT), and 1 mM phenylmethylsulfonyl fluoride] and disrupted by sonication. The lysate was centrifuged at 13,000*g* for 15 min at 4 °C, and the supernatant constituted the crude extract. Protein was quantified using the Bio-Rad Bradford protein assay with BSA as standard.

### *In Vitro* Assay of TMT Activity

TMT activity was assayed by methylation of γ-tocopherol to α-tocopherol using S-adenosyl methionine (SAM) as a methyl donor.^38^ The *in vitro* assay was performed as described by Gutbrod et al.,^39^ with modifications. Crude extracts (300 μg protein) were incubated with 50 mM HEPES (pH 7.8), 5 mM DTT, 50 μM SAM, and 50 μM γ-tocopherol in a total volume of 500 μL. The reaction mixture was incubated at 25 °C for 4 h and extracted with chloroform/methanol (2:1). After adding 300 mM NH_4_Ac and mixing, the chloroform phase was recovered, evaporated with N_2_, and resuspended in hexane for tocopherol quantification by HPLC.

### Tocopherol Analysis

Tocopherol analysis was carried out according to Narváez et al.^29^ Briefly, samples of 500 mg (DW) of lyophilized olive mesocarp tissue were extracted with hexane. An aliquot of the extracts was filtered and injected into an HPLC. The tocopherols were determined according to Abdallah et al.,^40^ using a Supersphere Si60 Lichrocart 250–4 HPLC cartridge (Merck) and eluted with hexane:2-propanol (99:1) at a flow rate of 1 mL/min. A fluorescence detector (Shimadzu RF535) was used, setting the excitation and emission wavelengths at 290 and 330 nm, respectively. The injection volume of the sample was 20 μL. Peaks of α-, β-, γ-, and δ-tocopherol were identified using a mixed standard solution of all four compounds. The quantitative evaluation was performed by external standardization using an α-tocopherol calibration curve. The data are presented as means ± SD of three biological replicates, each having three technical replicates.

## Results and Discussion

### cDNA Cloning and Sequence Analysis of Olive Tocopherol Methyltransferase Gene

A sequence homologous to the *Arabidopsis* *TMT* gene^19^ was found in the olive transcriptome^32^ and genome.^33^ Specific primers and the olive (cv Picual) fruit cDNA library were utilized for PCR amplification. A full-length cDNA (*OepTMT*) with 1188 bp was cloned, encoding a 373-amino-acid protein. The predicted protein displays a calculated molecular mass of 41.0 kDa, a p*I* value of 8.33, and shares 61% identity with *Arabidopsis* TMT (Figure 1).

**Figure 1:**
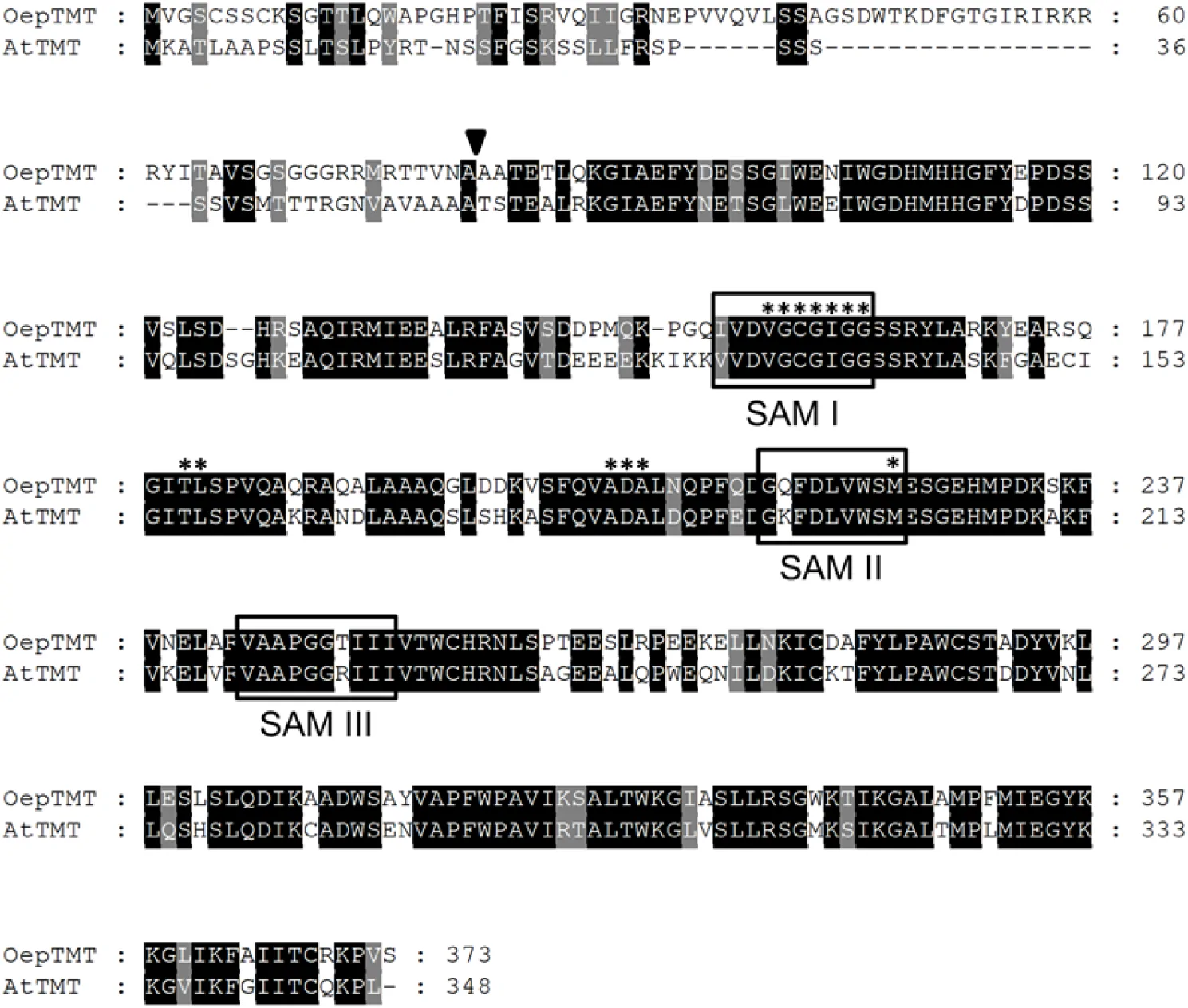
Comparison of the deduced amino acid sequences of olive and *Arabidopsis* (At1g64970) *TMT* genes. The sequences were aligned using the ClustalX program and displayed with GeneDoc. Identical and similar residues are shown on a background of black and gray, respectively. The putative cleavage site for a chloroplast transit peptide is denoted by an inverted triangle. The three SAM-conserved motifs and several conserved residues characteristic of *S*-adenosylmethionine-dependent methyltransferases are framed with a solid line and labeled with asterisks, respectively.

The conserved domain search classified the olive TMT within the *PLN02244* superfamily, with tocopherol O-methyltransferase activity, including the *S*-adenosylmethionine (SAM)-dependent methyltransferase domain (pfam08241) at positions 155–253. As shown in Figure 1, three highly conserved motifs (SAM I, II, and III) and several conserved residues characteristic of SAM-dependent methyltransferases were identified.^41,42^ On the other hand, the transmembrane prediction of the OepTMT protein, built on a hidden Markov model (TMHMM) analysis, was generated. However, no transmembrane domains were found in the olive TMT sequence, as reported for the TMT protein from rice.^43^

Concerning the subcellular localization, analysis of the OepTMT protein sequence with prediction software such as WoLF PSORT, ProtComp, or DeepLoc indicated a chloroplast localization. Furthermore, an N-terminal chloroplast signal peptide was detected by TargetP software, showing a predicted cleavage site after Ala at residue 81 (Figure 1). This chloroplast localization has been experimentally confirmed for TMT from other plants, such as sweetpotato^44^ or maize^45^ in tobacco leaves, and from alfalfa^46^ in *Arabidopsis* protoplasts.

To examine the genomic structure of the olive *TMT* gene, its cDNA nucleotide sequence was compared with the oleaster genome.^33^ The corresponding genomic sequence of the *TMT* homologue in the oleaster was found (Figure 2A). The olive *TMT* gene contained four introns, which is in line with the occurrence of three to five introns reported for *TMT* genes from other plants.

**Figure 2:**
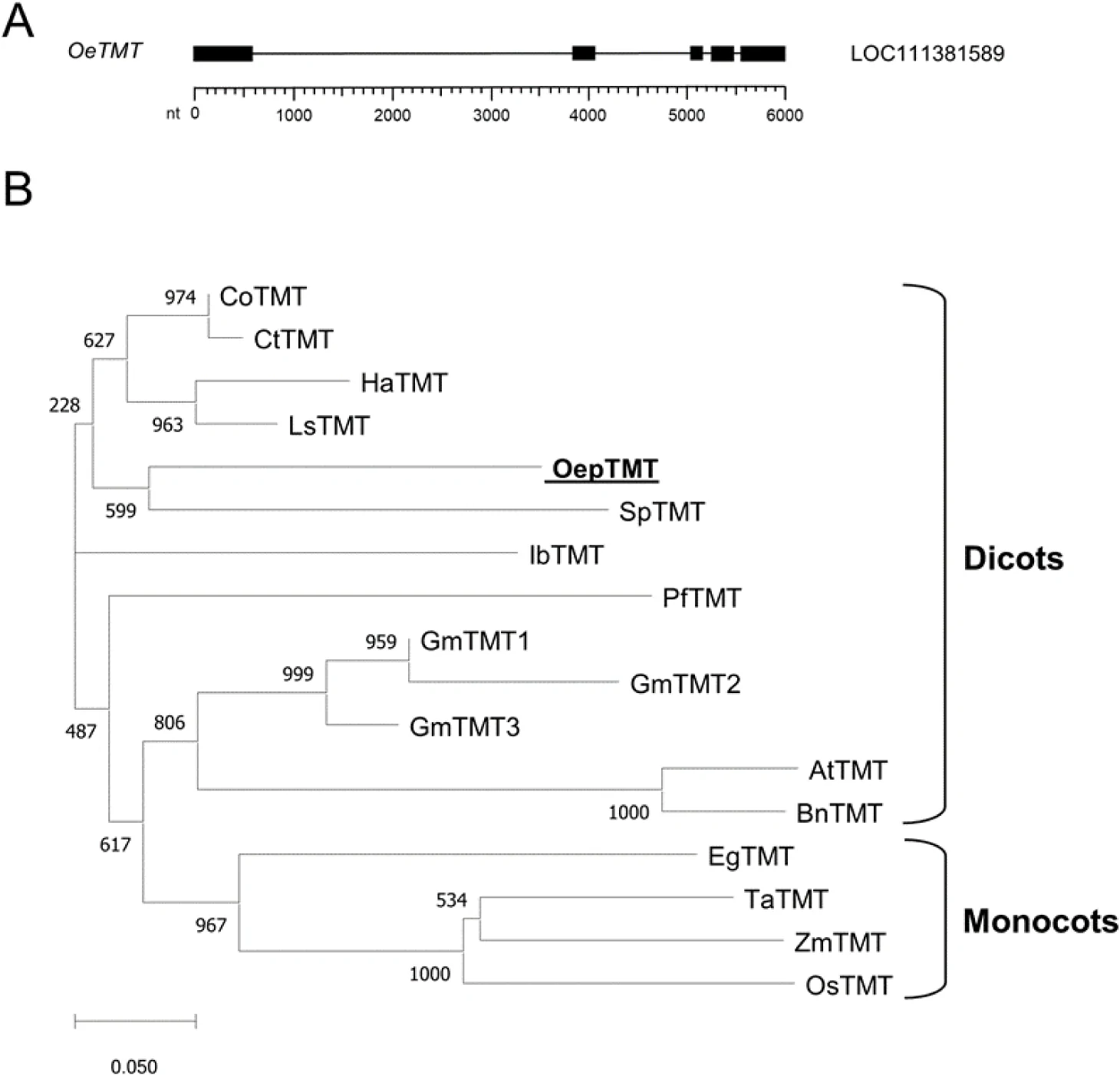
Structure of the *OeTMT* gene in the olive genome (A) and phylogenetic tree analysis of characterized plant TMT (B). In (A), black boxes denote exons in the open reading frame, and lines indicate introns. In (B), alignments were calculated with ClustalX, and the analysis was performed using the neighbor-joining method implemented in the Phylip package using Kimura’s correction for multiple substitutions and a 1000-bootstrap data set. TreeView was used to display the tree. Position of the olive TMT is in bold and underlined. Accession numbers of the different TMTs included in this analysis are indicated in the Supporting Information (Table S3).

To investigate the phylogenetic relationship of olive TMT, an unrooted phylogenetic tree with known and characterized plant TMTs (Table S3) was generated (Figure 2B). In accordance with previous reports,^47^ plant TMTs may be categorized into two distinct monocot- and dicot-specific clades, with OepTMT being situated in the branch along with other TMTs from dicot plants. It seems that TMTs evolved differently between dicots and monocots.

### Functional Expression of Olive Tocopherol Methyltransferase Gene in *Escherichia coli*

To verify the functional identity of the *OepTMT* gene, its open reading frame, lacking a putative N-terminal chloroplast transit peptide (Figure 1), was cloned into an IPTG-inducible *E. coli* expression vector. To express the recombinant protein, bacterial cells transformed with the olive *TMT* overexpression construct were grown at 22 °C for 20 h. Subsequently, crude extracts were employed to carry out the TMT enzyme activity assay. Proteins from crude extracts of *E. coli* cells expressing OepTMT converted γ-tocopherol into α-tocopherol, demonstrating that this recombinant protein possesses TMT activity *in vitro*, as well as confirming that the olive *TMT* gene encodes a functional TMT enzyme. Thus, the olive *TMT* gene codes for the enzyme which catalyzes the formation of α-tocopherol from γ-tocopherol in the presence of SAM and, therefore, has been functionally identified. Similar results have been reported for several plant recombinant TMT enzymes, such as those from *Arabidopsis* ^19^ and oil palm.^39^

### Tissue Specificity of Olive Tocopherol Methyltransferase Gene

To explore the physiological function of the olive *TMT* gene, we measured its transcript levels in diverse olive tissues from the Picual and Arbequina cultivars (Figure 3). *OeTMT* transcripts were detected in all studied tissues. In drupes and mesocarp, much higher expression levels were observed in the Arbequina cultivar compared to Picual. In the other two tissues from both cultivars, higher transcript levels were found in leaves in comparison to seeds, as has been reported in rice^48^ and alfalfa.^46^ On the contrary, the three TMT isoforms present in soybean showed lower expression levels in leaves compared to seeds.^49^ Additionally, all these results indicate a spatial regulation of the *TMT* gene in olive, given that it was differently expressed in all studied tissues.

**Figure 3:**
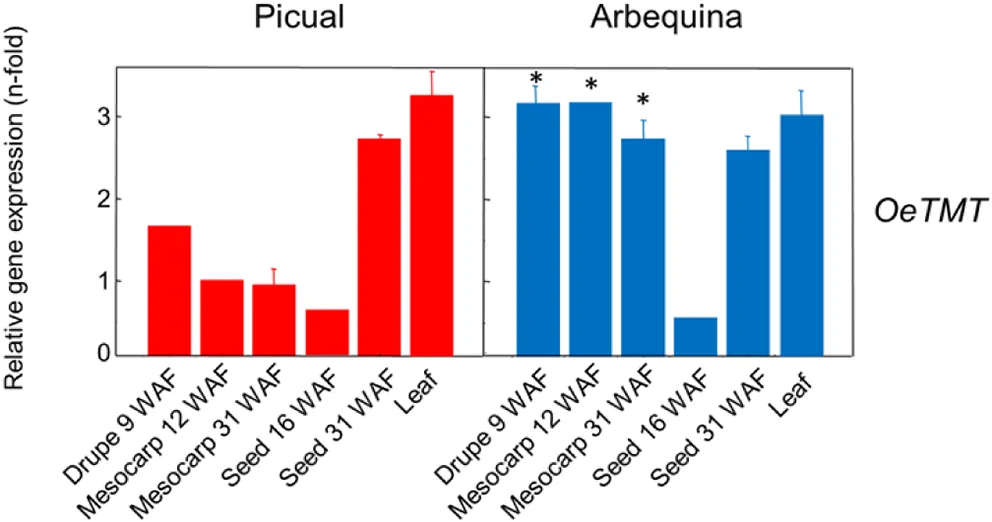
Relative expression levels of the olive *TMT* gene in different organs and tissues of Picual and Arbequina cultivars. The relative expression levels were determined by qRT-PCR. Data are presented as means ± SD of three biological replicates. *Indicates significantly different (*P* < 0.05) to “Picual” by two-way analysis of variance (ANOVA) with a Bonferroni posttest in “Arbequina” tissues.

### Developmental Expression of Tocopherol Methyltransferase Gene in the Olive Fruit in Relation to the α-Tocopherol Content

The olive *TMT* transcript levels in the olive fruit were analyzed in more detail. Specifically, seeds and mesocarp tissue from the cultivars Picual and Arbequina during olive fruit development and maturation were studied.

Regarding developing seeds (Figure S1), transcript analysis of the *TMT* gene showed an up-regulation in its expression levels through the whole developmental and maturation periods of olive fruit from both cultivars, displaying maximum values at 28 and 35 WAF for “Picual” and “Arbequina”, respectively. A comparable evolution pattern has been reported in both olive cultivars for *HPT* and *MPBQ MT* genes,^29^ which are implicated in the first two reactions of the committed pathway for tocopherol biosynthesis. The observed increase in the olive *TMT* gene expression during seed development has also been reported in soybean,^50^ oat,^51^ and blackberry.^52^

An analogous investigation was performed in “Picual” and “Arbequina” mesocarps (Figure 4). *TMT* expression declined all along olive fruit development and maturation (Figure 4B), mirroring the decreasing (α+β)-tocopherol/(γ+δ)-tocopherol ratio (Figure 4A). In the case of the cultivar Koroneiki, an up-regulation of the *TMT* gene was detected during the early stages of fruit maturation, and a diminution afterward.^53^ All these results point out that *TMT* gene expression seems to be temporally regulated in olive fruit. Furthermore, the *TMT* expression patterns observed in “Picual” and “Arbequina” mesocarps across development and maturation are parallel to those reported previously for *HPT* and *MPBQ MT* genes,^29^ suggesting a transcriptional coregulation of these three genes, which belong to the tocopherol biosynthetic pathway in the olive mesocarp. Regarding other fruits, an up-regulation of the *TMT* gene has been described during fruit ripening of different *Citrus* species,^54^ whereas a down-regulation has been reported in the case of sweet cherry fruits.^55^

**Figure 4:**
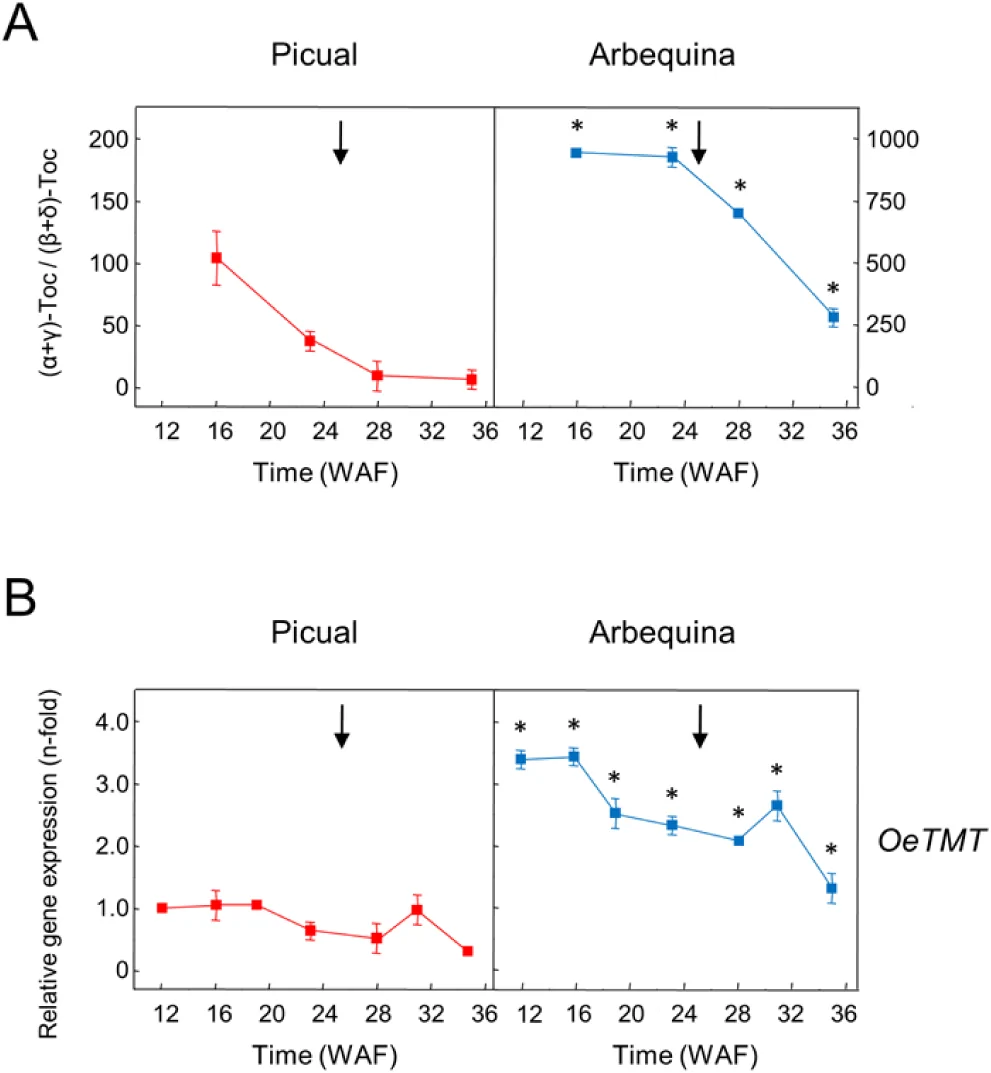
(α+β)-tocopherol/(γ+δ)-tocopherol ratio (A) and relative expression levels of the olive *TMT* gene (B) in the mesocarp tissue from Picual and Arbequina cultivars. The beginning of fruit ripening is denoted by an arrow. At the indicated stages, tocopherols were analyzed by HPLC, and the relative expression levels were determined by qRT-PCR. Data are presented as means ± SD of three biological replicates. *Indicates significantly different (*P* < 0.05) to “Picual” by two-way ANOVA with a Bonferroni posttest in “Arbequina”.

Our study was broadened to olive cultivars with low (190 and 285 mg kg^–1^ oil for “Klon-14” and “Abou Kanani”, respectively) or high (820 and 1610 mg kg^–1^ oil for “Piñonera” and “Dokkar”, respectively) tocopherol amounts in their oils.^24^ In this case, mesocarp tissue representing four distinct developmental and maturation fruit stages (Figure 5) was used. The observed decrease in the *OeTMT* transcript levels during olive mesocarp development and maturation in all studied cultivars (Figure 5B) was analogous to that detected for the (α+β)-tocopherol/(γ+δ)-tocopherol ratio (Figure 5A). These data suggest that TMT is the enzyme responsible for the biosynthesis of α- and β-tocopherols from γ- and δ-tocopherols, respectively, in olive mesocarp and, hence, in olive oil.

**Figure 5:**
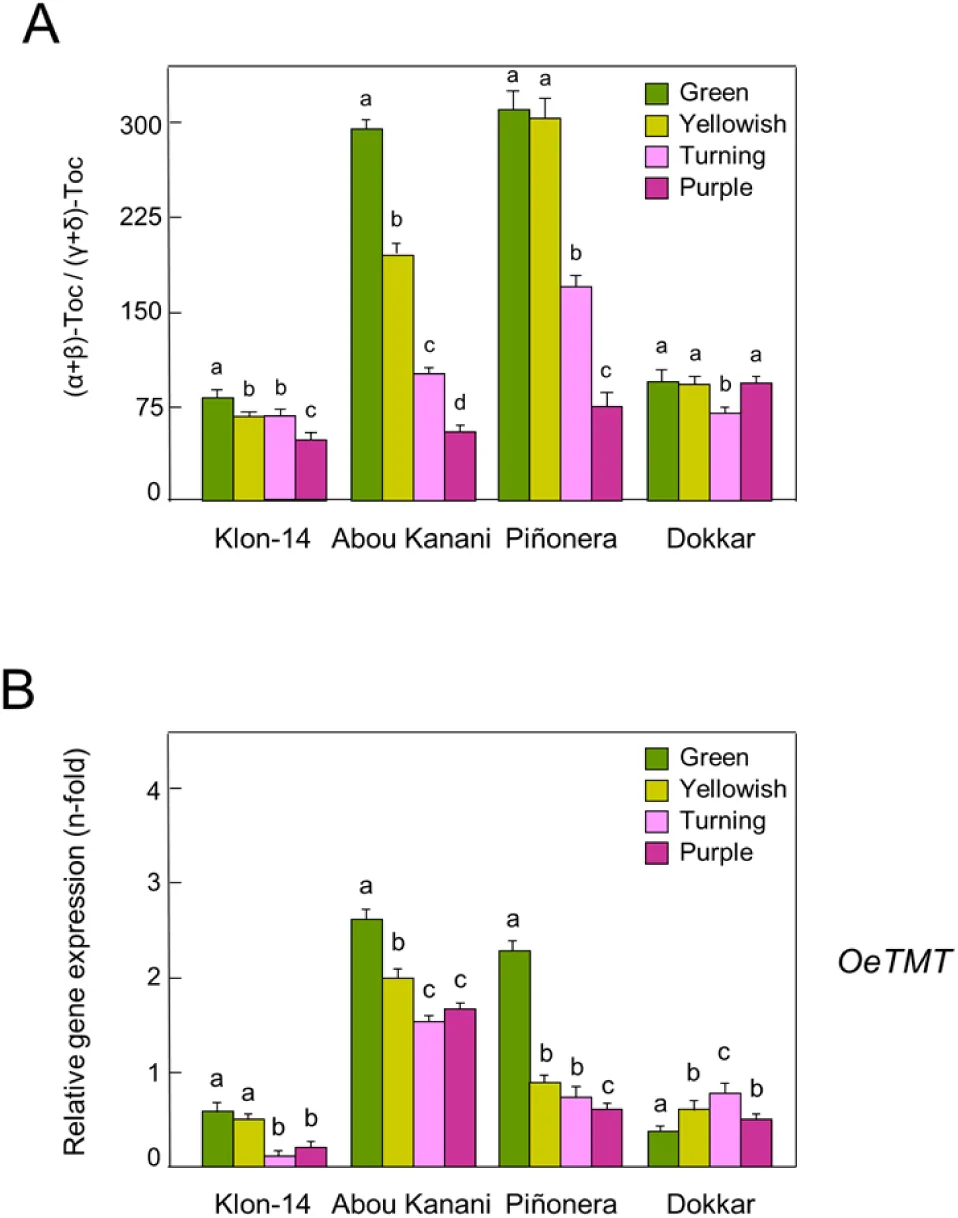
(α+β)-tocopherol/(γ+δ)-tocopherol ratio (A) and relative expression levels of olive *TMT* genes (B) in the mesocarp tissue from different olive cultivars. At the indicated stages, tocopherols were analyzed by HPLC, and the relative expression levels were determined by qRT-PCR. Data are presented as means ± SD of three biological replicates. Different letters denote significant differences (*P* < 0.05) for each gene and cultivar by one-way ANOVA followed by Tukey’s posttest for multiple comparisons.

Notably, the *OeTMT* expression levels were not lower in the cultivars characterized by a low tocopherol amount, such as in “Klon-14” and “Abou Kanani” (Figure 5A). Likewise, in “Piñonera” and “Dokkar”, with a high content of tocopherols, its transcript levels were not higher. These results indicate that TMT plays a significant role in determining the composition but not the total amount of tocopherols in olive mesocarp, as reported in *Arabidopsis* seeds.^19^

### Effect of Regulated Deficit Irrigation on Tocopherol Methyltransferase Gene Expression in the Olive Fruit Mesocarp

An increment in α-tocopherol content in response to drought has been described in the leaves of juvenile olive trees.^56^ In this study, higher expression levels of the *TMT* gene were found during fruit development and maturation in “Arbequina” mesocarp from olive fruits exposed to 60RDI and 30RDI treatments, which brought about significant water stress^30^ compared to the FI treatment (Figure 6).

**Figure 6:**
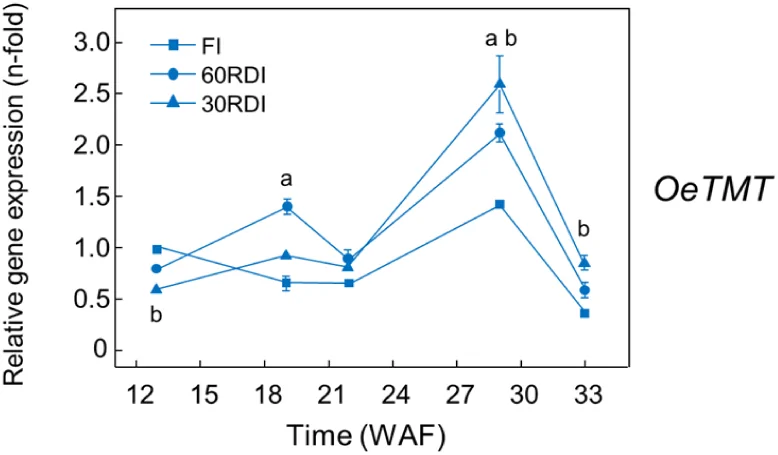
Effect of regulated deficit irrigation (RDI) treatments on the relative expression levels of the olive *TMT* gene in the “Arbequina” mesocarp during olive fruit development and maturation. The relative expression levels were determined by qRT-PCR, with the expression level of the *TMT* gene from full irrigation (FI) treatment at 13 WAF as the calibrator. Data are presented as means ± SD of three biological replicates. ^a^ and ^b^ indicate significantly different (*P* < 0.05) in 60RDI and 30RDI, respectively, to FI by two-way analysis of variance (ANOVA) with a Bonferroni posttest.

In a similar experiment, analogous increases in transcript levels were found by our group for *HPT* and *MPBQ MT* genes.^29^ Thus, the pathway for tocopherol biosynthesis seems to be transcriptionally up-regulated in olive mesocarp under water stress, with *OeHPT, OeMPBQ MT*, and *OeTMT* genes enhancing their transcript levels and producing an increase in the total amount of tocopherols in “Arbequina” mesocarp and, as a consequence, in their oil.^57^ In line with these data, the expression of genes implicated in tocopherol biosynthesis was increased as a response to lower rainfall during consecutive seasons in olives from the Koroneiki cultivar.^58^ Interestingly, increased expression levels of the gene encoding 4-hydroxyphenylpyruvate dioxygenase, which catalyzes the synthesis of homogentisate, a precursor of tocopherol biosynthesis, were observed in olive fruits from “Manzanilla” and “Picual” trees subjected to water stress.^59^ However, just one developmental stage was analyzed in that study.

An up-regulation of the *TMT* transcript levels has also been described in plant leaves subjected to drought, as in the case of lettuce^60^ and sweetpotato.^44^ Furthermore, it has been reported that overexpression of *TMT* in alfalfa confers increased drought tolerance, confirming the key role of α-tocopherol in the response to drought stress.^17^

### Transcriptional Regulation of Tocopherol Methyltransferase Gene in the Olive Fruit Mesocarp under Distinct Environmental Stresses

To assess how different abiotic stresses influence the expression of *OeTMT* in the “Picual” and “Arbequina” mesocarps, branches bearing turning-stage (28 WAF) olives were incubated for 24 h, changing the standard conditions (25 °C and 12-h light/12 h dark cycle) according to the stress to be studied. Under these standard conditions, no significant variation in *TMT* transcript levels was detected in the olive mesocarp (Figure S2).

An increase in the α-tocopherol content has been correlated with enhanced chilling tolerance in maize plants.^61^ Exposure of olive fruit to low temperature (15 °C) resulted in a transient elevation of *TMT* transcript levels in the mesocarp of the Picual cultivar, peaking at 6 h post-treatment (Figure 7A). On the contrary, in the “Arbequina” mesocarp, no significant changes in *TMT* expression levels were observed. In contrast to our data, a down-regulation of *TMT* transcript levels has been described in rice seedlings^47^ and *Chlamydomonas reinhardtii* cells^62^ exposed to low temperature, as well as in mandarin^63^ and grapefruit^64^ subjected to long-term storage under cold conditions. This discrepancy can be explained by the lower temperatures used in these studies (2–10 °C) compared with that employed in the present work (15 °C).

**Figure 7:**
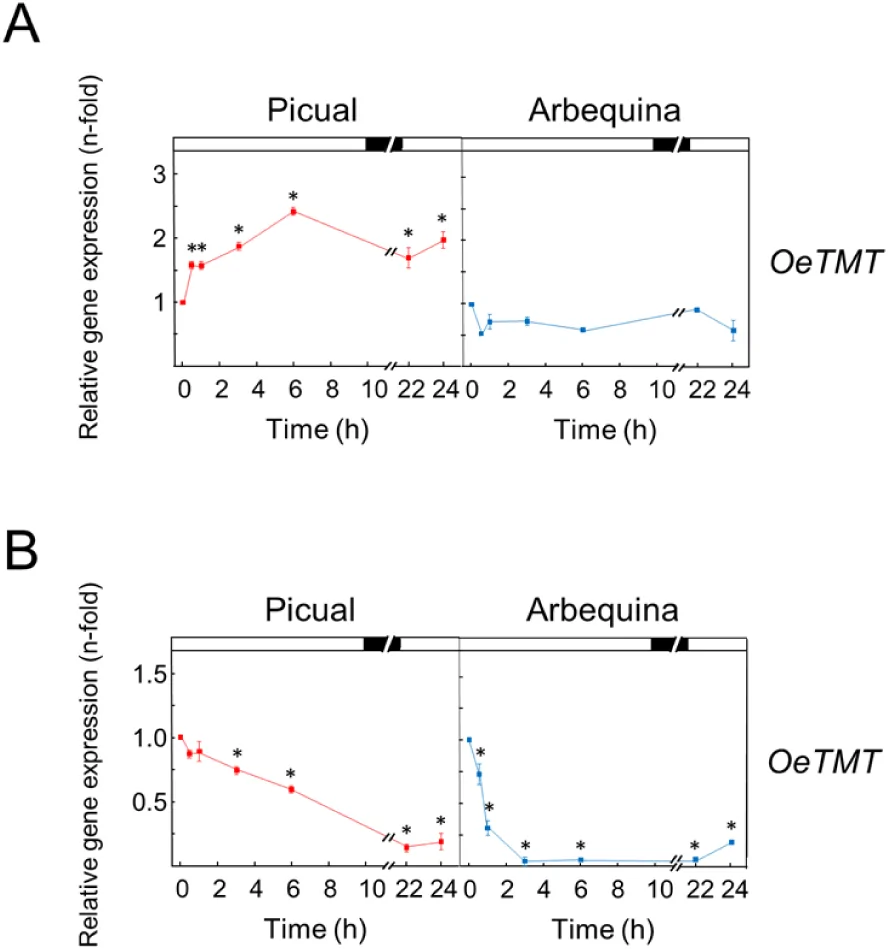
Effect of low (A) and high (B) temperature on the relative expression levels of the olive *TMT* gene in the mesocarp tissue from Picual and Arbequina cultivars. Olive tree branches with about 100 olive fruits (28 WAF) were incubated using standard conditions except that the temperature was 15 °C (A) or 35 °C (B). At the indicated times, the relative expression levels were determined by qRT-PCR, using the expression level of the *TMT* gene at zero time as a calibrator. Data are presented as means ± SD of three biological replicates. *Indicates significantly different (*P* < 0.05) to time 0 h by two-way ANOVA with a Bonferroni posttest. Boxes in the upper part indicate light (open) or dark (closed) periods.

When incubated at a high temperature (35 °C), both cultivars exhibited a marked decrease in TMT transcript abundance, eventually becoming undetectable (Figure 7B). *OeTMT* expression levels decreased progressively in “Picual”, whereas in “Arbequina” the decline was rapid and pronounced from the beginning of the treatment. In contrast, in *C. reinhardtii* cells incubated at a high temperature, no major changes in *TMT* expression levels were observed.^62^

The role of light in regulating α-tocopherol content has been documented in numerous plants, including *Arabidopsis*.^20^ To test the effect of darkness on *TMT* transcript abundance in “Picual” and “Arbequina” mesocarps, branches were kept in darkness at 25 °C for 24 h. Both cultivars exhibited a substantial reduction in *TMT* transcript levels, primarily within the first 3 to 6 h, with low expression maintained thereafter (Figure 8A). Similar down-regulation of the *TMT* gene under darkness has been described in rice shoots,^47^ tomato fruit,^65^ grapefruit,^64^ as well as in *C. reinhardtii* cells.^62^ These data indicate that the olive *TMT* gene is regulated by light.

**Figure 8:**
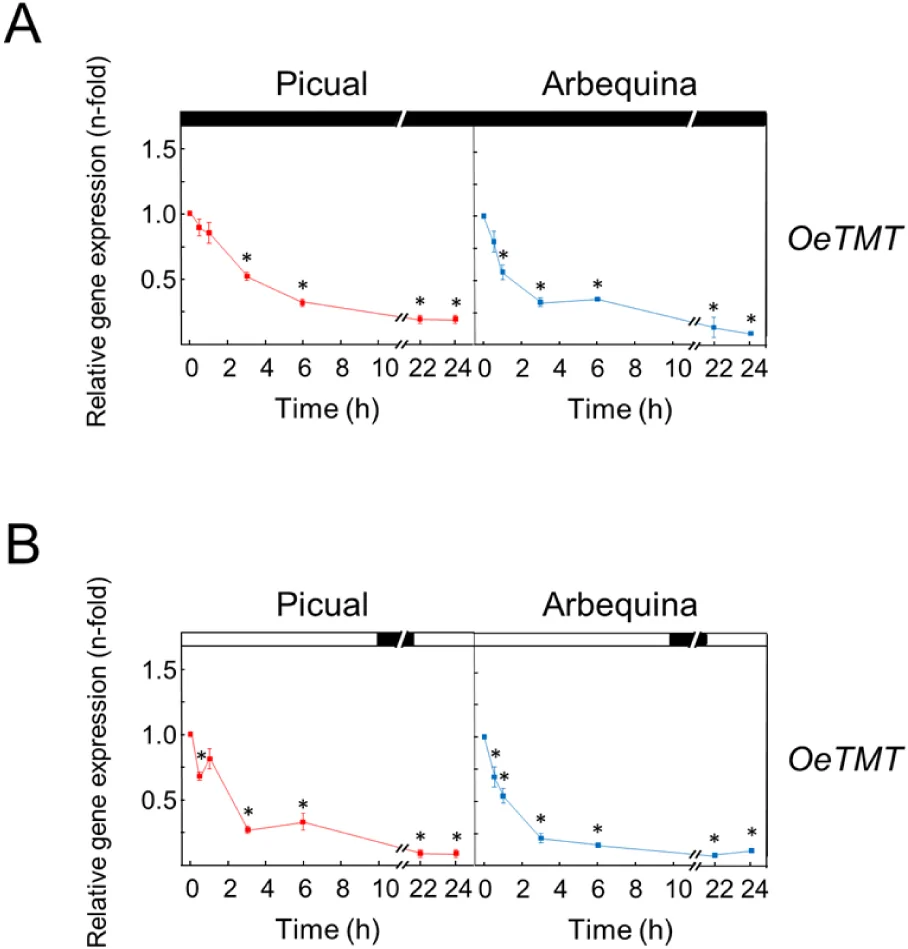
Effect of darkness (A) and wounding (B) on the relative expression levels of the olive *TMT* gene in the mesocarp tissue from Picual and Arbequina cultivars. Olive tree branches with about 100 olive fruits (28 WAF) were incubated using standard conditions, except that the olive fruits were incubated under darkness (A) or mechanically damaged (B). At the indicated times, the relative expression levels were determined by qRT-PCR, using the expression level of the *TMT* gene at zero time as a calibrator. Data are presented as means ± SD of three biological replicates. *Indicates significantly different (*P* < 0.05) to time 0 h by two-way ANOVA with a Bonferroni posttest. Boxes in the upper part indicate light (open) or dark (closed) periods.

Lastly, the impact of mechanical wounding on *TMT* expression was examined for the first time by subjecting “Picual” and “Arbequina” mesocarp tissues to damage and maintaining them under standard conditions. *OeTMT* transcript levels exhibited a fast and severe diminution in both cultivars (Figure 8B). In line with our results, a significant decrease in *TMT* transcript abundance was reported in *Arabidopsis* leaves infected with *Pseudomonas syringae*.^66^ However, no significant differences in *TMT* expression were observed in sweetpotato leaves in response to inoculation with the bacterial pathogen *Pectobacterium chrysanthemi* in comparison with the control.^44^ Notably, *Arabidopsis* *vte4* mutant displayed similar resistance to *Pseudomonas syringae* infection as wild-type plants but showed enhanced susceptibility to the fungal pathogen *Botrytis cinerea*.^67^

Overall, the expression patterns of olive *TMT* in the mesocarp under these environmental stresses closely resemble those of *HPT* and *MPBQ MT* genes,^29^ indicating a potential coordinated transcriptional regulation of genes of tocopherol biosynthesis in response to abiotic stresses.

In conclusion, the olive *TMT* gene has been cloned and characterized. Sequence analysis indicates that it encodes a TMT enzyme, and its biochemical function was confirmed *in vitro* using a bacterial expression system. Transcript profiling reveals a cultivar-dependent, spatial, and temporal regulation of *OeTMT* expression levels and, together with tocopherol analysis along fruit mesocarp development and maturation, also indicates that the *OeTMT* gene is implicated in the synthesis of the α-tocopherol existing in the fruit mesocarp and, consequently, in the VOO. Furthermore, *TMT* transcript levels in olive fruits are altered in response to water deficit, temperature, light, and mechanical wounding, suggesting a role for TMT in the response to abiotic stresses. These findings constitute a significant advance in the understanding of tocopherol biosynthesis regulation in olive fruit, informing optimal cultivation and harvesting conditions to enhance α-tocopherol content in the VOO. Additionally, this knowledge will facilitate the generation of molecular markers for breeding olive cultivars with increased vitamin E levels in the VOO.

## Supplementary Material



## Notes

Relative expression levels of the olive *TMT* gene in the seed tissue from Picual and Arbequina cultivars (Figure S1); the effect of incubation under standard conditions on the relative expression levels of the olive *TMT* gene in the mesocarp tissue from Picual and Arbequina cultivars (Figure S2); sequences of the primer pairs used for gene expression analysis by qRT-PCR (Table S1); sequences of the primer pairs used for amplification of olive TMT coding sequence for their functional expression in *E. coli* (Table S2), and accession numbers of the different TMTs included in the phylogenetic tree analysis (Table S3) (PDF)

## †

Departamento de Botánica y Fisiología Vegetal, Instituto de Hortofruticultura Subtropical y Mediterránea, Universidad de Málaga (IHSM-UMA-CSIC), Málaga, Spain

## ‡

Departamento de Bioquímica Vegetal y Biología Molecular/Instituto de Bioquímica Vegetal y Fotosíntesis (Universidad de Sevilla–CSIC), Avda. Américo Vespucio, 49, 41092, Sevilla, Spain.
