# Supplementary material for: Functional Characterization and Transcriptional Regulation of Olive Tocopherol Methyltransferase Responsible for the Vitamin E Content of Virgin Olive Oil
Source: J Agric Food Chem. 2026 Aug 24;74(35):27757–66. doi: 10.1021/acs.jafc.6c07768 (PMC13573554; doi:10.1021/acs.jafc.6c07768)
Supplement: Supplementary file 1 [file jf6c07768_si_001.pdf]

SUPPORTING INFORMATION

Functional Characterization and Transcriptional Regulation of Olive Tocopherol Methyltransferase Responsible for the Vitamin E Content of the Virgin Olive Oil

Cristina Muñoz-Ocaña, Isabel Narváez[a], M. Dolores Sicardo, M. Luisa Hernández[b], Wenceslao Moreda and José M. Martínez-Rivas*

Department of Biochemistry and Molecular Biology of Plant Products, Instituto de la Grasa (IG), CSIC, 41013-Seville, Spain.

*Corresponding author: J.M. Martínez-Rivas.

Email: mrivas@ig.csic.es

[a]Present address: Departamento de Botánica y Fisiología Vegetal, Instituto de Hortofruticultura Subtropical y Mediterránea, Universidad de Málaga (IHSM-UMA-CSIC), Málaga, Spain

[b]Present address: Departamento de Bioquímica Vegetal y Biología Molecular/Instituto de Bioquímica Vegetal y Fotosíntesis (Universidad de Sevilla-CSIC). Avda. Américo Vespucio, 49. 41092, Sevilla, Spain.

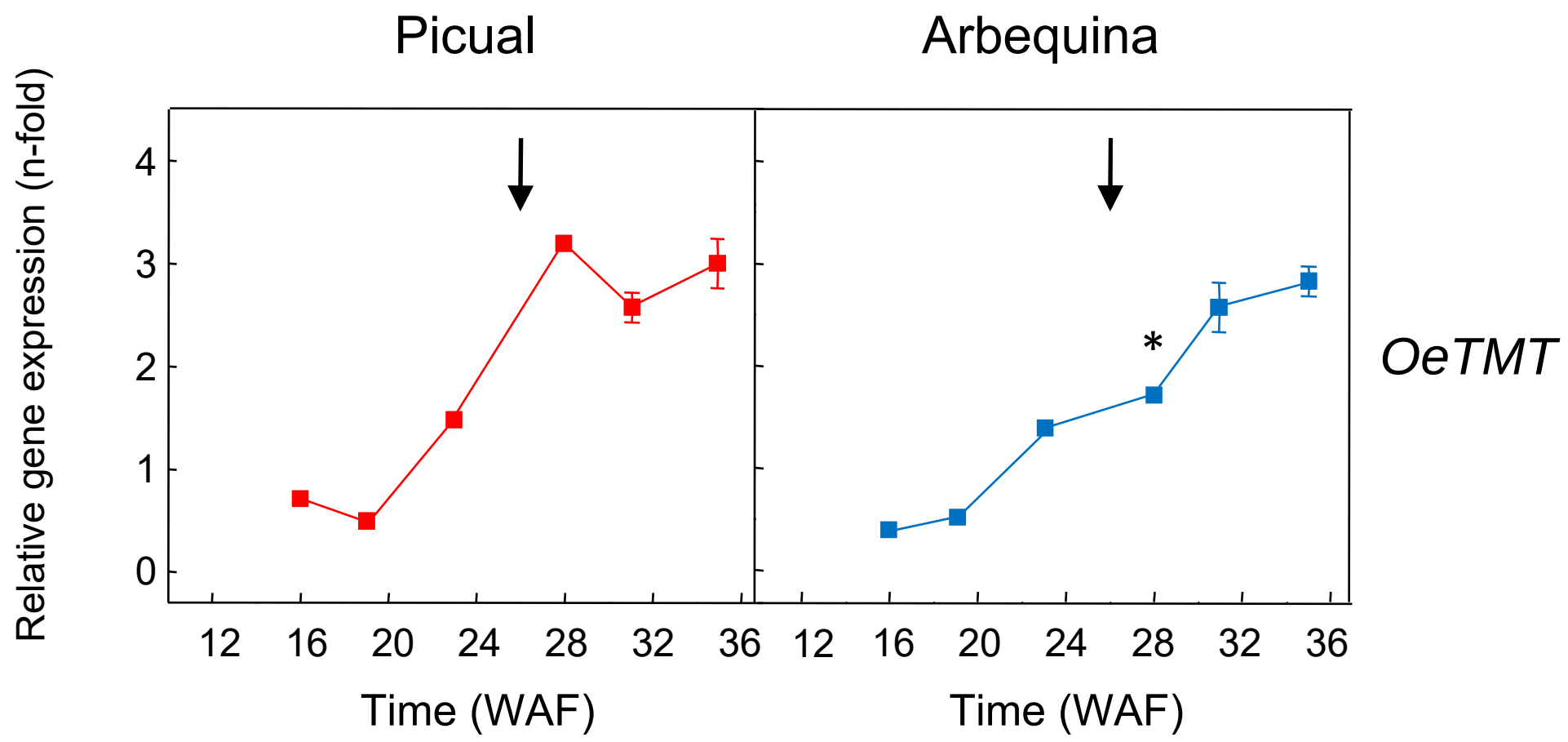

Figure S1. Relative expression levels of olive *TMT* gene in the seed tissue from Picual and Arbequina cultivars. The beginning of fruit ripening corresponding to the appearance of a pink-purple colour is denoted by an arrow. At the indicated stages, the relative expression levels were determined by qRT-PCR as described under section “Materials and Methods”. Data are presented as means ± SD of three biological replicates. *Indicates significantly different ($P<0.05$) to ‘Picual’ by two-way ANOVA with a Bonferroni posttest in ‘Arbequina’.

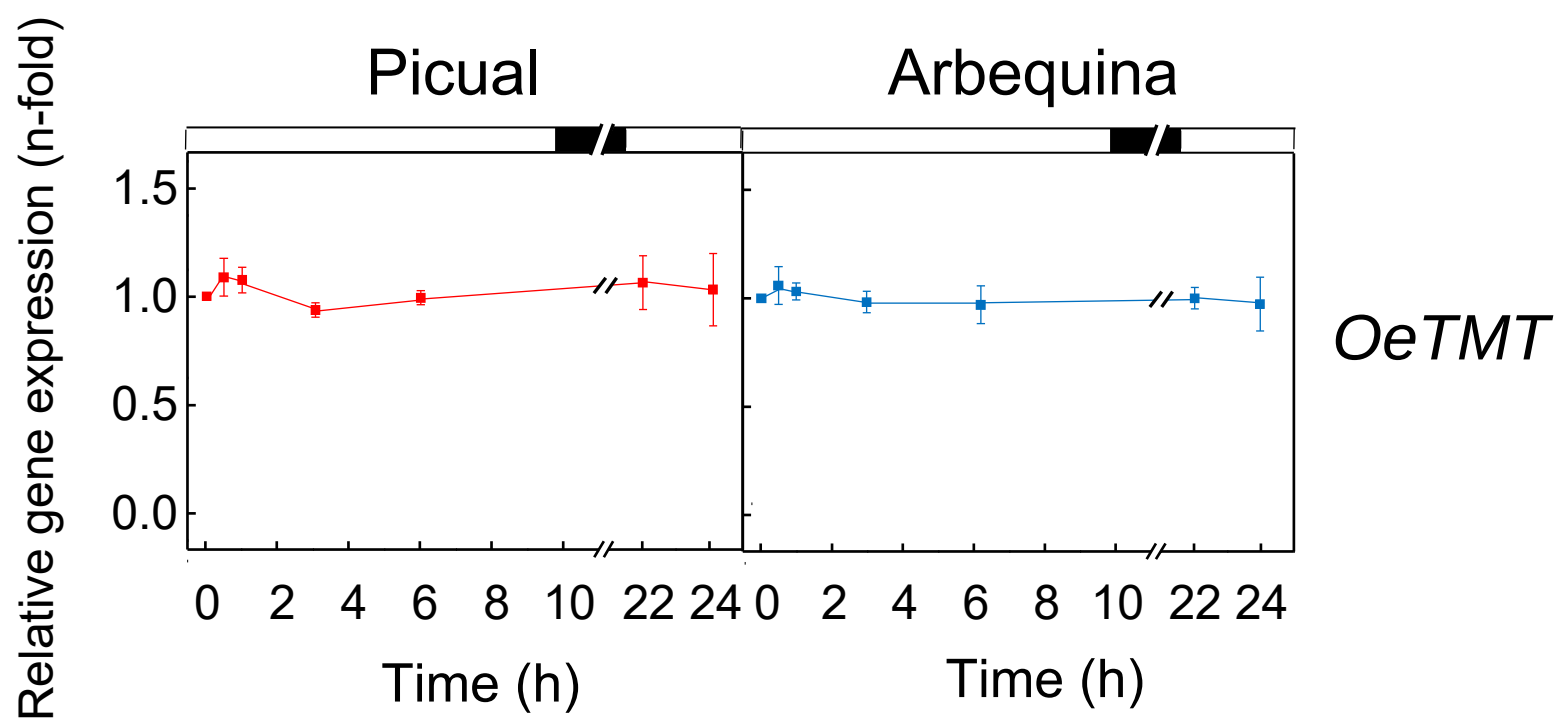

Figure S2. Effect of incubation under the standard conditions on the relative expression levels of olive *TMT* gene in the mesocarp tissue from Picual and Arbequina cultivars. Branches with about 100 olive fruit (28 WAF) were incubated using standard conditions. At the indicated times, relative expressions levels were determined by qRT-PCR as described in Materials and methods, using the expression level of the corresponding gene at zero time as calibrator. Data are presented as means ± SD of three biological replicates. Boxes in the upper part indicate light (open) or dark (closed) periods.

Table S1. Sequences of the primers pairs used for gene expression analysis by qRT-PCR in the present study.

Gene	Sequence	Amplicon size (bp)
OeTMT	Forward: 5'- AGTCAGCATTGACATGGAAGG -3'<br>Reverse: 5'- AATCTCATGACTCGGGCTTTC -3'	156
OeUBQ2	Forward: 5'-AATGAAGTCTGTCTCTCCTTTGG-3'<br>Reverse: 5'-AAGGGAAATCCCATCAACG-3'	132

Table S2. Sequences of the primers pairs used for amplification of olive *TMT* coding sequence for their functional expression in *E. coli*.

Gene	Sequence	Restriction enzyme
OepTMT	Forward: 5'- GATCTATTCGAA**T**GCGGCGACGGAGACTTTGCA -3'	*Bst*BI
	Reverse: 5'- ATCTGTACATCACTCCTGATTATGTACT -3'	*Bsr*GI

Restriction sites are underlined. The nucleotide incorporated to maintain the reading frame of the genes is marked in bold.

Table S3. Accession numbers of the different TMT included in the phylogenetic tree analysis.

PLANT	GENE	ACCESSION NUMBER
Arabidopsis thaliana	*AtTMT*	AF104220
Brassica napus	*BnTMT*	EU637012
Carthamus oxycanthus	*CoTMT*	JX035783
Carthamus tinctorius	*CtTMT*	JX035782
Elaeis guineensis	*EgTMT*	XM_010907927
Glycine max	*GmTMT1*	MK416203
Glycine max	*GmTMT2*	MK416204
Glycine max	*GmTMT3*	MK416205
Helianthus annuus	*HaTMT*	DQ229830
Ipomoea batatas	*IbTMT*	KP306527
Lactuca sativa	*LsTMT*	FJ975047
Olea europaea	*OepTMT*	PX970942
Oryza sativa	*OsTMT*	AK061746
Perilla frutescens	*PfTMT*	AF213481
Solanum pennelli	*SpTMT*	HQ014376
Triticum aestivum	*TaTMT*	AJ920394
Zea mays	*ZmTMT*	AJ634706
